# Human Monkeypox: Oral Implications and Recommendations for Oral Screening and Infection Control in Dental Practice

**DOI:** 10.3390/jpm12122000

**Published:** 2022-12-02

**Authors:** Massimo Amato, Federica Di Spirito, Giovanni Boccia, Domenico Fornino, Francesco D’Ambrosio, Francesco De Caro

**Affiliations:** Department of Medicine, Surgery and Dentistry, University of Salerno, Via S. Allende, Baronissi, 84081 Salerno, Italy

**Keywords:** monkeypox, oral lesions, oral signs, oral manifestations, dental practice, dental office, dental setting, infection control, prevention, transmission, cross-infections

## Abstract

The World Health Organization declared the spread of the human monkeypox virus (MPXV) an “emerging threat of moderate health concern” on 23 June 2022. Although about 20,000 cases of Monkeypox (MPX) were recorded in Europe and more than 28,000 in the United States from May to October 2022, their number is still small compared to the number of dental patients treated annually. Therefore, the likelihood of oral healthcare workers encountering an MPX case is relatively low in not endemic regions. In addition, MPX-positive individuals are considered contagious only during the prodromal or acute phase. However, the exact shedding and transmission routes of MPX and the associated risk of transmission in the dental setting remain unclear. Moreover, infected subjects whose disease is confined to the head and neck may require oral and dental care because they complain of lymphadenopathy involving the cervical lymph nodes. Furthermore, MPX lesions may first appear in the oral cavity or perioral area. Therefore, given the recent spread of MPXV in non-endemic areas where dentists are not used to considering this disease in the differential diagnosis and taking appropriate preventive measures, all oral healthcare providers nowadays should be aware of the oral presentation of MPX for adequate oral screening and appropriate preventive measures for infection control in the dental practice.

## 1. Introduction

Monkeypox (MPX) is a zoonotic disease caused by the monkeypox virus (MPXV), which belongs to the genus Orthopoxvirus of the Poxviridae family [1,2]. Poxviruses are a highly heterogeneous family of enveloped double-stranded DNA viruses that multiply in infected cells [3,4]. Two genetic clades of the monkeypox virus have been characterized: Clade I in Central Africa (Congo Basin) and Clade II in West Africa, and they cause similar clinical conditions.

Human monkeypox was first identified in 1970 in the Democratic Republic of Congo in a nine-month-old baby. The child had a smallpox-like disease from which the MPXV had been isolated [5,6,7,8,9]. Since then, most cases have been reported from the rural rainforest regions of the Congo Basin. The first patient zero in Nigeria was reported in 1971 [10]. After recognition as a human pathogen, 59 cases of monkeypox have been reported in humans between 1970 and 1980, with a mortality rate of 17%. Since 1980, several thousand human cases of monkeypox have been reported in 15 different countries, including 11 African countries [10]. Sporadic MPX cases have been subsequently diagnosed in the United Kingdom, the United States, Israel, and Singapore. An outbreak of monkeypox with a concomitant chickenpox outbreak was found in the Democratic Republic of the Congo in 1996, which could lead to a change in transmission dynamics [10].

Nigeria has suffered from a large epidemic since 2017. Moreover, in recent years, MPXV spread in several cities outside the virus-endemic regions among individuals who had not traveled to Africa, thus suggesting person-to-person transmission and drawing the attention of the health authorities. However, MPXV diffusion was rarely reported in geographic regions with low MPX prevalence till April 2022.

On 6 May 2022, the first case of monkeypox in the UK was confirmed in a man who had traveled from Nigeria and had started developing symptoms as early as April 29 while still in the African country [11]. In Italy, the first case was confirmed on 20 May 2022. Since early May 2022, more than 3000 MPX cases have been detected in more than 50 countries, prompting the World Health Organization (WHO) to declare MPX an “emerging threat of moderate health concern” on 23 June 2022 [12]. Most of the reported cases in non-endemic countries involve men who are gay, bisexual, or who have had sexual intercourse with other men. Unlike previous sporadic cases, the ongoing MPXV epidemic has affected human communities that do not have travel links to virus-endemic regions [13].

The number of confirmed cases of MPX has continued to increase in non-virus-endemic areas, reaching 22,000 cases in approximately 88 countries in North America, Europe, and Australia [14], and leading the World Health Organization to declare MPX as a global health emergency and to raise the alert for this infection to the highest level, on 22 July 2022 [9]. Altogether, about 20,000 MPX cases were registered in Europe and more than 28,000 in the United States from May to October 2022 [15,16].

Considering the insufficient dissemination of knowledge about MPX risk factors, clinical presentation and outcomes, and viral transmission routes and clearance [12], and the recent spread in non-endemic areas [14,15,16] where dentists are not used to considering this disease in differential diagnosis and taking appropriate preventive measures [17,18], the present study aimed to narratively review currently available data on the epidemiologic, clinical, and preventive characteristics of MPX; to increase oral healthcare providers’ awareness of the oral implications of MPX, appropriate oral screening, and integrated oral-systemic care; and to provide evidence-based recommendations for MPXV infection control in the dental office.

## 2. Epidemiology

Phylogenetic analyses suggest that the virus has been circulating undetected outside areas where it is endemic for some time [12].

Beyond changes in viral biology, the smallpox immunity declining, the relaxation of preventive measures against SARS-2, the resumption of international travel, and sexual interactions associated with large gatherings may subtend the current global outbreak of human MPXV infections [15].

The high probability of sexual transmission was supported by the finding of primary genital, anal, and oral mucosal lesions that may represent the site of inoculation. In addition, 98% of cases were men who are gay, bisexual, or have sex with men, and 41% were living with HIV infection and had a history of sexually transmitted infections suggesting increased transmission through sexual networks [19]. However, it remains to be investigated whether semen can transmit the infection, as it is still ignored whether the viral DNA detected in these samples could be replicated; ascertained transmission routes are detailed below.

### 2.1. Animal-to-Human Transmission

MPX is a zoonotic virus transmitted from animals to humans through a bite or a scratch of infected animals, either through respiratory droplets or preferentially direct contact with organic material in the mucocutaneous lesions or body fluids from an infected animal or contaminated material [17,20,21]. Consequently, sleeping outdoors or visiting the forest in the tropics, potentially increasing exposure to animals, are considered risk factors enhancing the probability of MPXV transmission from animals to humans [22,23].

Consumption of inadequately cooked meat from an infected animal has also been suggested as a possible additional risk factor [17,24].

### 2.2. Human-to-Human Transmission

The main entry sites for MPX are open wounds, non-intact skin, mucous membranes, and inhalation [25,26]. Therefore, the human-to-human transmission of MPXV, similarly to animal-to-human transmission, occurs by direct contact with the material in lesions or airborne droplets [27].

In detail, the infection spreads through large respiratory droplets and typically requires prolonged close contact, unlike SARS-CoV-2 infection, which, on the other hand, can spread through even small droplets [28,29]. However, similarly to other airborne infections, the transmission depends on the presence of respiratory protective equipment, infectious dose, duration of exposure, and environmental temperature, humidity, and ventilation [26,29].

Noteworthy, some authors have shown that MPX can remain infectious in aerosols for several hours [30,31].

MPXV can also be transmitted via body fluids such as blood and probably semen, while transmission through saliva has not yet been ascertained [32,33]. However, high viral loads were detected in some rectal swabs and in urine, fecal, semen, and saliva samples at various time points after diagnosis. Noteworthy, MPXV DNA was detected in all saliva samples from the 12 cases studied by Peiró-Mestres, showing intermittent shedding [19], and was reported as detectable in saliva until 76 days after laboratory confirmation in 1 case [34].

Sexual contact has been documented in about 90% of cases [35]. However, it is not yet clear whether the virus is sexually transmitted or spreads through direct contact with skin lesions or via body fluids such as saliva and respiratory secretions [36], specifically via droplets and contaminated surfaces [37], and via infectious aerosols, with an infected person during sexual activity [38,39].

Coherently, also drinking or eating from the same bowl and sleeping in the same room increases the risk of contracting the virus [40,41,42].

Moreover, fetal deaths have been noticed, thus highlighting a potential vertical transmission has also been hypothesized [12,43].

To date, the human-to-human transmission alone is thought to be insufficient to sustain outbreaks of MPXV in humans, requiring repeated reintroduction of the virus from the wild. However, it is also suggested that viral evolution [44], altered host behavior, and ecological changes may be responsible for the persistence of MPXV in humans and the increase in primary human cases [45].

## 3. Case Definition and Diagnostic Procedures

Suspected case identification largely relies on an accurate case definition and clinical examination [46].

### 3.1. Case Definition

According to the WHO definition, a confirmed case is “A person with laboratory-confirmed monkeypox virus infection by detection of unique sequences of viral DNA by real-time polymerase chain reaction (PCR)^3^ and/or sequencing” [47].

On the contrary, a discarded case is defined as a “suspected or probable case for which laboratory testing of lesion fluid, skin specimens or crusts by PCR and/or sequencing is negative for MPXV” [47].

**Figure 1:**
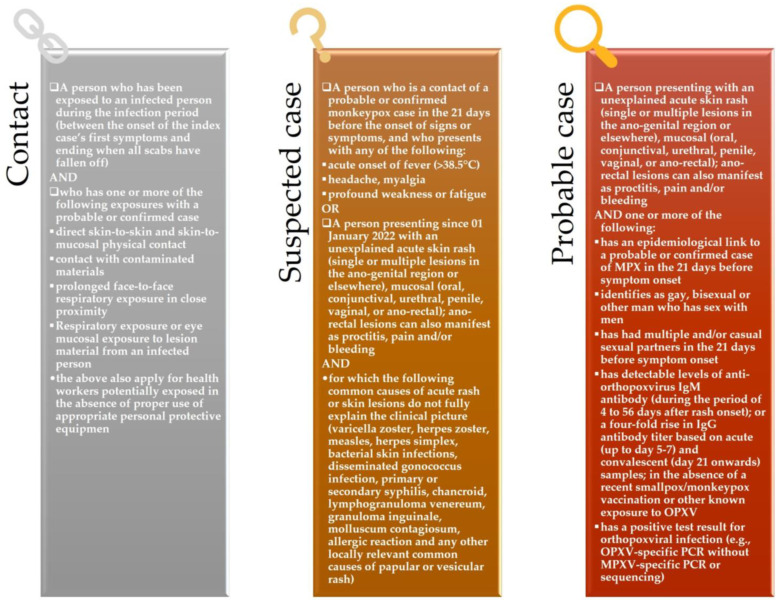
WHO definitions of MPX contact, suspected case, and probable case.

Contact, suspected, and probable case WHO definitions are illustrated in Figure 1.

As per currently accepted UK Health Security Agency guidelines [48,49], MPX diagnosis is probable in subjects with clinical signs and symptoms of the disease and having the following characteristics:

- Had contact with a person with probable or confirmed monkeypox in the 21 days before the onset of symptoms;
- Have traveled to West or Central Africa in the 21 days preceding the onset of the symptoms;

or

- Are gay, bisexual, and other men who have sex with men.

However, it has also been proposed that since sexually transmitted infections were reported concurrently in nearly 30% of MPX cases, monkeypox diagnosis should also be considered in at-risk subjects presenting with typical symptoms of sexually transmitted infections [12].

Moreover, international case definitions may need to include additional symptoms in the future, such as anogenital and oral mucosal lesions [12,46], which may be the first manifestation.

### 3.2. Diagnostic Procedures

MPX should be suspected in patients with fever and incipient skin rash, especially if lymphadenopathy is also present, especially in endemic countries.

In addition, unusual acute rashes, mainly when associated with MPX typical systemic symptoms, should be appropriately investigated and tested in non-high-risk individuals as well [12].

The World Health Organization recommends polymerase chain reaction (PCR) testing to detect MPXV in swabs from skin, genital, and oral mucosal lesions, and, less commonly, in blood. Anal or rectal swabs should also be considered in patients with anal pain or proctitis [12].

These tests are only practicable in public health laboratories in developed countries, as commercial tests are not currently available [17], except for a rapid point-of-care diagnostic (Tetracore Orthopox BioThreat Alert^®^) developed in 2003 [50,51].

Conversely, antibody and antigen detection methods do not provide specific confirmation for MPX due to serologic cross-reactivity of orthopoxviruses and possible other positive results from previous smallpox vaccinations [17]. Moreover, serum antibodies are detected approximately two weeks after exposure, coincident with the appearance of oral or skin lesions [52].

#### Monkeypox Saliva-Based Testing

Laboratory analysis showed strong agreement between tests based on saliva and swabs from mucocutaneous lesions [53].

Among the potential advantages of saliva-based testing is that earlier identification of cases before the lesions appear, with earlier isolation and treatment, may reduce the risk of infection.

Considering also that anogenital lesions have consistently occurred in the current outbreak [37,54] and that collecting anogenital specimens may be more challenging compared with saliva specimens, particularly if existing testing centers for COVID-19 are adapted, saliva testing could facilitate and improve the availability and approachability of MPXV testing.

## 4. General Clinical Features

MPX incubation period commonly ranges between 7 and 14 days to 21 days [12], while prodromal symptoms lasting from 2 to 4 days [55,56,57] include fever, malaise, headache, and lymphadenopathy [46].

**Figure 2:**
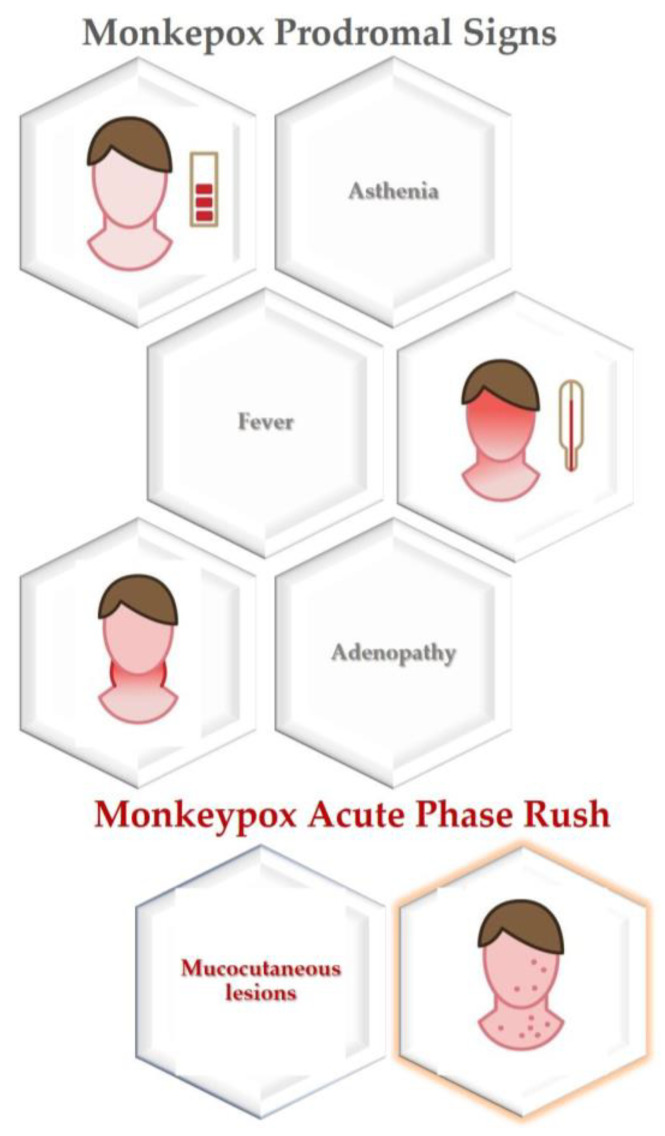
Monkeypox general clinical presenting features.

In the subsequent acute phase, the initial presenting feature is mucocutaneous involvement showing a very polymorphic appearance. The most common presentation is a single or multiple lesion(s), mainly occurring on the anogenital area, body (trunk or limbs), face, or a combination of these sites, which become more numerous over time, along with variable systemic signs. The median time between the first mucocutaneous lesion and the development of additional ones ranges between 2 and 11 days. Of note, the clinical presentation described is similar in individuals with and without HIV [12] (Figure 2).

Cutaneous involvement is characterized by single to thousands of lesions with centrifugal distribution, evolving in about 12 days from maculae to papules, vesicles, pustules that become umbilicated, and erosions, concurrently present at various stages [55,57]. Skin vesicle ruptures may leave a black crust that eventually falls off [58].

Mucocutaneous lesions were observed in 95% of individuals [12].

Noteworthy, perioral mucocutaneous lesions may be among the first MPX manifestation [12], presenting as papules, which evolve into blisters and ulcers [33] around the lips, chin, and nose [26].

Nonetheless, the most frequently affected sites were the (73%) anogenital area; (55%) trunk, arms, or legs; (25%) face; and (10%) palms and soles [12]. In detail, the involvement of the anorectal mucosa was frequently associated with diarrhea and/or pain and/or tenesmus and/or proctitis, differently combined among each other, while lesions affecting oral mucosa and tonsils were often accompanied by odynophagia, epiglottitis, or pharyngitis [12].

Profound lymphadenopathy is the unique feature clinically discriminating between MPX and chickenpox, smallpox, and chickenpox [12,43,57]; thus, laboratory testing is essential [57].

Gastrointestinal symptoms and hematologic abnormalities are also common [12,17,59,60].

Fever usually reduces within 3 days following the onset of the mucocutaneous lesions, which generally resolve in 2 to 4 weeks [54,57]. Indeed, MPX is usually self-limiting and resolves within approximately one month [26], although viral positivity has been found up to 21 days after symptom onset [12] and recent evidence found the viral DNA in saliva more than 70 days after positivity confirmation [57].

Illness complications that can occur, especially for children and immunocompromised patients, as well as pregnant women, include pain, and secondary infections of the lungs, brain, and cornea, beyond psychological symptoms [12,57].

## 5. Oral Lesions

### 5.1. Macroscopic Features

The first lesions may occur in the mucous membranes and specifically in the oral mucosa, showing a maculopapular pattern with lesions ranging from 2 to 5 mm in diameter [12,61]. Subsequently, oral lesions may, spreading centrifugally, show various distribution patterns affecting the face, genital area, palms, and soles [12] and becoming generalized in most cases [61,62].

Oral ulcerations of the tongue, tonsils, and buccal mucosa are estimated to be the most common oral lesions, possibly associated with the impaired ability of patients to drink and eat and consequent dehydration and malnutrition [12,26].

**Figure 3:**
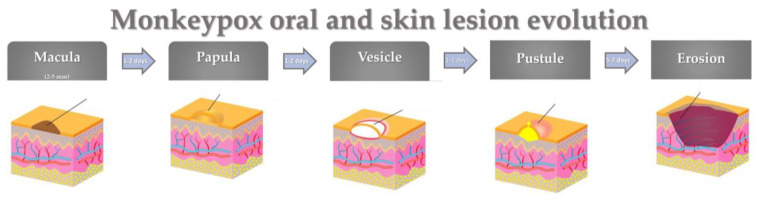
Monkeypox oral and skin lesion evolution.

Similar to skin lesions, the oral lesions typically progress through the maculopapular, vesicular, pustular, and erosive phases over a period of 14 to 21 days (Figure 3), potentially resulting in a depigmented scar [61,63].

### 5.2. Microscopic Features

Since MPX mucocutaneous lesions may macroscopically resemble chickenpox and smallpox ones [8] and differ markedly between anti-smallpox vaccinated and non-vaccinated subjects [8], microscopic features enable the final diagnosis [61].

#### 5.2.1. Histopathology

Acute poxvirus infections and oral lesions due to Herpesviridae share similar histologic features, including marked spongiosis, ballooning degeneration of keratinocytes, acute dermal inflammation, and edema [61].

Diagnostic histopathologic features, hardly described, clearly reflect the clinical progression of the lesions and can be detected at the vesicular and pustular phases.

In the vesicular stage, the uninvolved peri-lesional epithelium shows spongiosis, moderate acanthosis, and exocytosis of neutrophils with neutrophilic debris and lymphocytes; a band-like inflammatory infiltrate of lymphocytes and eosinophils is located at the epithelial-connective junction at the vesicle’s base. The vesicular-bullous lesion shows ballooning degeneration of epithelial cells interspersed with a mixed inflammatory cell infiltrate of lymphocytes, neutrophils, and rare eosinophils. The involvement of basal cells with balloon-like degeneration gives the bulla a subepithelial component. In the center of the bulla, degenerated epitheliocytes are seen in all layers, along with rare multinucleated epithelial cells whose nuclei have normal morphology and show no viral cytopathic changes [61].

In the pustular phase, only a few viable epithelial cells are detectable. Some of them have eosinophilic nucleoli mimicking intranuclear inclusions; others exhibit the viral cytopathic effect manifested as a “frosted glass” in the central region of the nucleus, with the nuclear contents pushed to the periphery and forming a basophilic “halo.” Eosinophilic, homogeneous, spherical intracytoplasmic inclusions are also seen within affected epithelial cells. Lymphocytes, eosinophils, and neutrophils are intermingled within the epithelium, dispersed between collagen fibers in the underlying connective tissue, and show perivascular and perieccrine distribution [61].

#### 5.2.2. Immunohistochemistry

Immunohistochemistry helps distinguish between Herpesviridae and smallpox viruses related lesions.

Anti-vaccinia antibodies are detected in all epithelial cells within the vesicle, including multinucleated cells and cells with conspicuous nucleoli, but not in the peri-lesional epithelium, and highlight the spherical intracytoplasmic inclusion bodies within the affected epitheliocytes [61,64].

#### 5.2.3. Electron Microscopy

When observed at the electron microscope, epitheliocytes show a multitude of mature and immature virions assembled in the cytoplasm [61,65].

### 5.3. Putative Pathogenic Mechanisms

The degenerative changes and the detection of viral antigens in the oral mucosa progress through the outer layers, thus suggesting an initial infection of the basal layer [32] as also observed in the skin samples, where the basal layer also appeared to be the initial infection site.

Such degenerative changes in the MPX vesicles are probably the result of rapid hyperplasia of the cells of the Malpighian layer and balloon cell degeneration.

The necrotic changes within the epitheliocytes are due to apoptosis. Coherently, significant apoptotic labeling is detectable in the areas where the lesions occur, consistent with the distribution of viral antigens detected by immunohistochemistry [32].

In addition, Langerhans cells, which are known to be susceptible to vaccinia virus infection after exposure, presumably serve as vehicles for initial viral transport as they migrate from exposed skin or mucous membranes to regional lymph nodes [65].

In a similar way, Langerhans cells are likely to become infected with MPXV and serve as vehicles for transporting the virus to regional lymph nodes, resulting in severe lymphadenopathy and causing MPX leukocyte-associated viremia [61,64].

### 5.4. Differential Diagnosis

The progression of the oral mucosal lesions may not follow exactly the evolution pattern described, further complicating the identification of oral monkeypox [26]. Therefore, a thorough history should be taken, and the advice of the local health authority should be sought for possible or probable MPX cases [26].

In addition, erosions and ulcers of the tonsils may be mistaken for tonsillitis [12], while those of the tongue may be confused with syphilis [57]; if isolated, a traumatic origin should be excluded, rather considering the infective nature of the lesion in case of fever and lymphadenopathy [26].

Varicella zoster virus infection, particularly the maculopapular skin lesions of chickenpox, should be considered primarily as a differential diagnosis and distinguished by dermatomal distribution, the tendency to coalescence, and lack of umbilication as opposed to MPX lesions [26]. In addition, pre-eruptive fever, lymphadenopathy, and slower maturation of the mucocutaneous lesions may be the most essential clinical clues to distinguish monkeypox from varicella [61,66].

## 6. Oral Screening

Although patients with acute malaise or a widespread pox-like rash are more likely to present to physicians than to dentists, those with disease limited to the head and neck may present to dentists first complaining of lymphadenopathy, which may involve the cervical lymph nodes.

Moreover, patients with a limited rash may have lesions only in the oral cavity or perioral area [26].

Furthermore, MPX primary lesions may occur in the oropharynx before cutaneous involvement.

Therefore, in patients with an unexplained rash and one or more symptoms typical of monkeypox [12], oral healthcare providers should consider MPXV among the possible causes [26].

Opportunistic oral screening should be advocated in high-risk individuals. Accordingly, oral health care providers may intercept enlarged lymph nodes, oral manifestations such as erythematous tongue or oral ulcers, and perioral lesions as part of routine examination, thus contributing to early diagnosis and timely containment of MPX [46].

In addition, in individuals with oral manifestations of sexually transmitted infections, especially with the erosive phenotype (i.e., oral syphilis [57]), further investigation may be recommended to rule out concurrent infection with MPXV to avoid underdiagnosis and misdiagnosis of monkeypox, as has been suggested for genital mucosa [12,19].

Potentially overlapping oral HIV-related lesions [67,68] should also be considered with special attention, as nearly half of the cases diagnosed with MPX in the current outbreak were HIV-positive [19].

**Figure 4:**
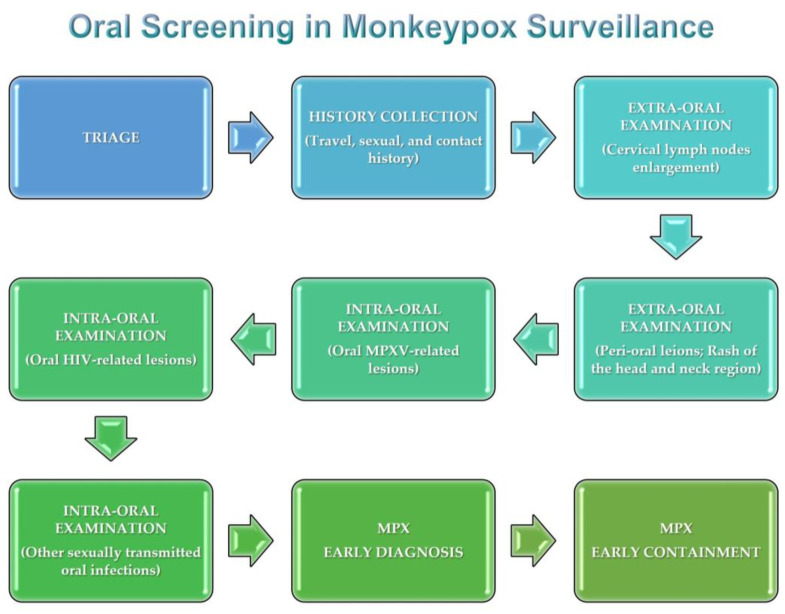
Oral screening in MPX surveillance.

Given the recent spread of MPX in non-endemic areas where dentists are not used to considering this disease in the differential diagnosis [69], all oral health care professionals should be made aware of the oral lesions of MPX and the manifestations in the head and neck region. With this in mind, targeted education about MPX oral screening, illustrated in Figure 4 should be conducted.

## 7. Infection Prevention and Control for Monkeypox in Dental Practice

### 7.1. Counteracting Infectious Diseases in Dentistry: Old, New, and Reemerging Viruses

Dentistry has historically adapted its infection prevention practices to both emerging [68] and reemerging [70] infectious diseases, as demonstrated during the human immunodeficiency virus (HIV) outbreak in the 1980s [26,68,71] and more recently during the COVID-19 pandemic [72,73,74,75]. The SARS-CoV-2 outbreak forced oral healthcare providers to get used to prevention protocols and precautions against airborne and aerosol-transmitted pathogens [76,77,78,79], in addition to those long used against bloodborne infections [67,68].

Nevertheless, similar to other healthcare settings [9,17,80], there is still poor evidence on the risk of MPXV transmission during dental procedures, and overall in dental offices and on appropriate infection control measures [81,82].

Research on infection prevention and control should be continuously encouraged and supported to ensure safe healthcare. In turn, oral healthcare workers should adhere to available guidelines and recommendations, which may be updated as the MPX epidemic develops and in anticipation of other possible future outbreaks [29].

### 7.2. Risk of Monkeypox Transmission in Dental Practice

Although the ongoing MPX outbreak is of major public health importance, the number of cases is small compared to the millions of dental patients treated annually [26]. Consequently, in geographic regions with low MPX prevalence, the likelihood of oral healthcare workers encountering an MPX case remains proportionally low, in contrast to countries with high transmission rates in the general population, where the risk of viral transmission and cross-infection in the dental setting may be increased [26].

Moreover, MPX-positive subjects are considered potentially infectious during the prodromal or acute phase, hypothetically reducing the likelihood of viral transmission to oral healthcare providers. However, the exact MPX shedding and the related risk of transmission in dental settings remain ignored [12,35,83].

Nevertheless, a large percentage of the population visits dental offices, so some patients with monkeypox might seek dental care during times of increased risk of infection in the general population. In addition, reinfected individuals can also spread the infection even if they are asymptomatic carriers. Therefore, oral care providers need to be aware not only of the clinical presentation of the disease but also of the associated preventive measures for infection control in dental settings [26,29,82].

Given the MPX transmission routes, all healthcare workers are at increased risk of infection from close and prolonged contact with patients [36,37,84]. Although it is unlikely that dental staff would knowingly or unknowingly come into contact with a case of MPX in the dental setting, the main risk of transmission is likely to be through direct contact with skin lesions or clothing that has been in contact with lesions [26].

Moreover, oral healthcare providers may be at additional risk [17,26,85] because they have close contact with patients for prolonged periods, and dental procedures may generate infected droplets and aerosols [26,29,82]. Indeed, infected fluids from perioral or oral lesions containing MPXV or from saliva and blood can enter the environment through direct contact and droplets.

In addition, MPXV remains infectious in aerosols for several hours [38], as also found in animal models [66], so although aerosolization is not the main route of transmission, it can be considered an important route of transmission in dental settings, further increasing the risk of occupational exposure for dental personnel and cross-infections in dental settings [26].

Furthermore, as previously demonstrated for herpesviruses and SARS-CoV-2, periodontal pockets may serve as MPXV reservoirs, along with salivary glands, and may, thus, indirectly affect the risk of viral transmission and reinfections in the dental setting [74].

### 7.3. Prevention and Control of Monkeypox Infection in Dental Settings

Basic principles of infection control are currently considered able to contain MPX spread, through timely suspected, probable, and confirmed case identification, isolation, contact tracing, and surveillance during the viral incubation period, and the use of personal protective equipment (PPE) by healthcare workers [17].

The main transmission route remains that of contact with the lesion, so it is crucial to implement that series of precautions to control standard and contact infections when treating patients with symptoms of MPX [83,86].

Since the airway is a possible transmission route, all air infection control protocols must be implemented through the use of N95 masks by all dental personnel present and other personal protective equipment (PPE) when operating a person infected with MPX or even in the only case of suspicion [17].

MPX can be transmitted from person to person, not only by direct contact and airborne droplets but also via aerosols. Dentists use rotary instruments with water cooling systems, including ultrasonic scalers and handpieces, and air-water syringes in their clinical practice, generating an aerosol of water droplets, saliva, blood, and microorganisms, possibly including MPX viruses. Consequently, the same precautions to combat the spread of SARS-CoV-2 infection should be taken [87].

Noteworthy, no specific antiseptic used as a mouthwash has been recommended [73,88,89,90].

**Figure 5:**
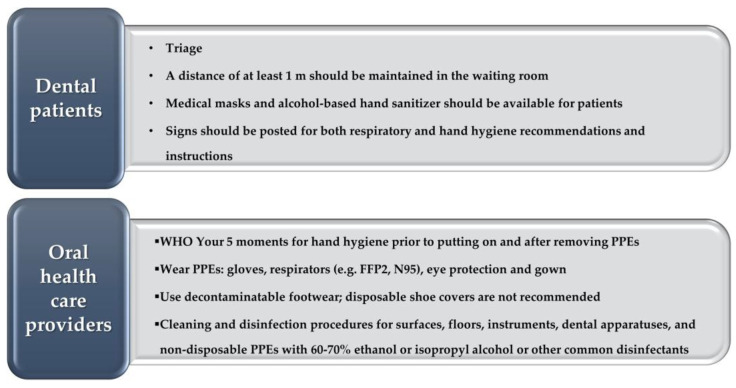
Synthetic recommendations for Prevention and control of Monkeypox infection in dental settings.

The WHO and UK guidelines [39,91] for the control of MPX have been jointly synthesized in Figure 5.

## 8. Monkeypox and Dental Patients’ Clinical Management

### 8.1. Triage

A comprehensive history is the first step in identifying patients who may have monkeypox. Patients who have traveled to areas where MPX is endemic or belong to high-risk groups are more likely to have contracted the virus.

Similarly, patients who report headache, muscle pain, fatigue, fever, and lymphadenopathy should either be treated with caution or deferred in non-urgent cases. These symptoms should be observed longer than with COVID-19 to rule out or confirm monkeypox infection, which prolongs patient monitoring.

If MPXV infection is suspected, the patient should wear a surgical mask and be asked to go home to isolate and await further advice, and the oral healthcare provider(s) should inform the local health protection team [26].

Accordingly, healthcare workers with suspicious symptoms or a history of contact should stay away from work [83].

### 8.2. Ergonomics

Similar to the recommendations and guidelines introduced to combat SARS-CoV-2 transmission, it is advised to avoid the presence of accompanying persons and reduce the number of people in the waiting room and the waiting time [72,73].

Operative and nonoperative rooms should be organized to ensure adequate ventilation and appropriate timing for cleaning and disinfection procedures [72,73].

Cleaning and disinfection of work areas should preferably be performed with a disinfectant containing 60–70% ethanol or isopropyl alcohol, following the manufacturer’s instructions regarding product concentration, contact time, and application methods [19,89].

Healed patients should be recalled after at least one month, although excretion of MPXV via saliva may take longer [19,72,92,93].

Teledentistry may also contribute to MPXV infection control, especially for those patients with contact history and MPX probable, very probable, and confirmed cases.

In detail, teleconsultations may aid patients’ triage and preliminary needs evaluation. Telediagnosis may also allow MPXV-related oral lesions screening, as well as teleassistance for dental and oral traumas and emergencies, and telemonitoring of ongoing treatments may be beneficial [94,95,96,97,98,99].

Mobile health applications and tools may assist in post-operative MPX case management, biofilm control, and oral, dental, and periodontal health literacy and prevention [97,100].

Moreover, teledentistry platforms may support inter-professional collaboration between physicians and oral healthcare workers in the assessment of case-specific benefit/risk ratio in delaying oral and dental care till complete healing from MPX and may optimize inter-disciplinary case evaluation in modulating treatment planning of complex dental and periodontal cases in relation to illness confirmation and progression [73], while reducing oral healthcare worker exposure to the virus [101,102].

Online education, focused on MPX oral screening, potential implications for MPXV salivary diagnosis, and preventive infection control measures, should, nevertheless, be implemented in non-virus-endemic areas to increase oral health care providers’ knowledge and preparedness and constantly updated under epidemic evolution and new evidence acquisition.

## 9. Monkeypox Suspected and Confirmed Dental Patients’ Management

Possible, probable, very probable, or confirmed MPX cases should avoid close contact with others until complete lesions healing [17]. Consequently, routine and elective dental treatments should not be performed in suspected and confirmed MPX cases.

However, for urgent oral and dental care procedures, dental patients with suspected or confirmed MPX should be referred to a public facility with adequately trained and equipped healthcare personnel [72,73]. If this is not possible, suspected or confirmed MPX cases requiring access to an outpatient clinic should be placed in a single room and should not be treated by immunocompromised or pregnant women [72,73].

As previously suggested, dental patients with suspected or confirmed MPX should preferably be treated as the last patient of the day, in an isolated room, under negative pressure if possible, performing procedures associated with a lower risk of viral transmission, i.e., using manual rather than aerosol-generating instruments, and overall favoring faster and more solution-oriented and less complex approaches, thereby reducing contact time, complications, and additional operative sessions [73].

### 9.1. Monkeypox Suspected Case Management in Dental Practice

When treating MPX suspected cases, the UK public health agencies recommended healthcare providers the use of [39]:

➢ Gloves;
➢ Eye protection;
➢ Water-repellent surgical mask (“replaced with an FFP3 respirator and eye protection in case the patient has a lower respiratory tract infection with cough” [39]);
➢ Gowns.

### 9.2. Monkeypox Confirmed Case Management in Dental Practice

When treating MPX confirmed cases, the UK public health agencies recommended healthcare providers the use of [39]:

➢ Gloves;
➢ Eye protection;
➢ FFP3-approved respirator;
➢ Disposable long-sleeved, water-repellent gown.

## 10. MPX Treatment and Vaccination

### 10.1. Monkeypox Treatment

MPX is a self-limiting infection; therefore, treatment is essentially symptomatic [17].

No specific but effective antiviral agents are available, including tecovirimate, cidofovir, and brincidofovir, which can be prescribed for patients with comorbidities [103], as well as immunoglobulin vaccinia (6000 UI/kg to 24,000 UI/kg) for passive immunization [104].

### 10.2. Monkeypox Vaccination

MPXV and smallpox are closely related; therefore, smallpox vaccination also provides some protection against monkeypox [105]. However, since smallpox was eradicated in 1980 by large-scale vaccination, population immunity to orthopoxviruses has declined, and MPXV is the major orthopoxvirus pathogen in humans [17,50,52,106].

A third-generation smallpox vaccine with an attenuated vaccinia virus is available in the U.S. and the United Kingdom.

In addition, smallpox vaccines are used for post-exposure prophylaxis in contacts of confirmed cases. They effectively prevent infection when administered immediately after exposure [107]; alternatively, vaccine immunoglobulin can be administered [26,29,108].

The clinical course of MPX and the epidemiologic situation do not currently compel a mass vaccination campaign. Moreover, considering the likely insufficient availability of vaccine doses, vaccination should be proposed for high-risk individuals, thus comprising: laboratory personnel with possible direct exposure to orthopoxvirus, and gay, transsexual, bisexual, and other men who have sex with men, with a recent history (in the past three months) of multiple sexual partners and/or participation in sexual encounters in clubs/saunas, and/or with sexually transmitted infections (in the past year), and/or with a habit of combining sexual acts with the use of chemical drugs (“chemsex”) [109,110].

Depending on epidemiological trends, physicians and oral healthcare providers may also be considered in the future.

## 11. Conclusions

Human Monkeypox (MPX) is an emerging virus with potential implications for oral care and dental practice.

Common symptoms of monkeypox include mucocutaneous lesions, often affecting the oral cavity and perioral tissues first, and cervical lymphadenopathy. Therefore, opportunistic oral screening of at-risk individuals should be advocated as part of the routine examination to contribute to early diagnosis and timely containment of MPX.

Although the current MPX outbreak is of great public health importance, the number of cases is small compared to the millions of dental patients treated annually. However, the precise shedding of MPX and the associated risk of transmission in the dental setting continue to be ignored, and dental patients with MPX may seek oral and dental care during periods of increased infectiousness in the general population. Therefore, oral and dental care providers need to be aware not only of the clinical presentation of the disease but also of the associated prevention measures.

Conventional infection prevention methods, that adhere to standard, contact, droplet, and aerosol infection control measures, are likely sufficient to reduce the risk of monkeypox in dental practice.

Future studies should highlight the possible existence of a pre/a-symptomatic MPX phase that, similar to asymptomatic carriers, could extend viral transmission into the dental setting, clarify routes of transmission and viral shedding, and increase knowledge of MPX risk factors, complications, and coinfection rates. These findings could be important for identifying high-risk individuals and for early diagnosis and containment.

Further research is needed to understand the potential role of MPX saliva testing and overall prevention measures in reducing the risk of emerging and reemerging viral infectious diseases in dental practice.

The reported findings and associated recommendations may be updated as new evidence becomes available and as the epidemic evolves.

## Data Availability

Data supporting reported results can be found on Scopus, Web of Science databases, and PubMed/MEDLINE.
